# Cardio-renal and cardio-hepatic interactions predict cardiovascular events in elderly patients with heart failure

**DOI:** 10.1371/journal.pone.0241003

**Published:** 2020-10-23

**Authors:** Takahiro Okano, Hirohiko Motoki, Masatoshi Minamisawa, Kazuhiro Kimura, Masafumi Kanai, Koji Yoshie, Satoko Higuchi, Tatsuya Saigusa, Soichiro Ebisawa, Ayako Okada, Morio Shoda, Koichiro Kuwahara

**Affiliations:** Department of Cardiovascular Medicine, Shinshu University School of Medicine, Matsumoto, Nagano, Japan; Universita degli Studi di Napoli Federico II, ITALY

## Abstract

**Background:**

The composite Model for End-Stage Liver Disease Excluding International Normalized Ratio Score (MELD-XI) is a novel tool to evaluate cardio-renal and cardio-hepatic interactions in patients with advanced heart failure (HF). However, its prognostic ability remains unclear in elderly HF patients.

**Methods and results:**

From July 2014 to July 2018, patients hospitalized for HF were prospectively recruited at 16 centers. Clinical features, laboratory findings, and echocardiography results were assessed prior to discharge. Cardiovascular (CV) death and HF re-hospitalization were recorded. Of the 676 patients enrolled, 264 (39.1%) experienced CV events throughout a 1-year median follow-up period. Patients with high MELD-XI were predominantly male and had a higher prevalence of NYHA III/IV, history of HF admission, hyperuricemia, ventricular tachycardia, anemia, and ischemic heart disease. In Kaplan-Meyer analysis, patients with higher MELD-XI (≥11) scores showed a worse prognosis than did those with lower (<11) scores (log-rank p≤0.001). Multivariate Cox proportional hazards testing revealed MELD-XI as an independent predictor of CV events (HR: 1.033, 95% CI: 1.006–1.061, p = 0.015) after adjusting for age, gender, body mass index, NYHA III/IV, prior HF hospitalization, systolic blood pressure, ischemic etiology, ventricular tachycardia, anemia, BNP, and left ventricular ejection fraction.

**Conclusions:**

Cardio-renal and cardio-hepatic interactions predicted CV events in aged HF patients.

## Introduction

Patients with heart failure (HF) are at significant risk for cardiovascular (CV) events such as death, myocardial infarction, stroke, and hospitalization for worsening HF, especially those >60 years old [1]. The elderly often have multiple comorbidities, including hypertension, atrial fibrillation (AF), renal dysfunction, chronic obstructive pulmonary disease, and anemia, all of which aggravate HF and affect prognosis [2]. Although sufficient multi-disciplinary interventions for HF are available, some elderly patients with HF suffer non-cardiac death. Thus, a predictive model for CV events is needed to distinguish patients at high risk for CV events from those at risk for non-cardiac death based on clinical risk factors.

Multi-organ dysfunction diminishes prognosis and renal dysfunction is a common adverse outcome predictor in HF [3]. Liver dysfunction has also been implicated in HF outcome; cardio-hepatic syndrome is considered a disorder characterized by the development of congestive hepatopathy and subsequent cirrhosis in advanced HF patients [4]. As a composite of serum bilirubin, creatinine, and international normalized ratio (INR), the Model for End-Stage Liver Disease (MELD) is an accurate metric of the degree of liver disease and a useful decision-making tool for liver transplantation [5]. MELD can be employed in advanced HF to assess for left ventricular device or heart transplantation requirement and as a prognostic indicator of death [6,7].

Recently, MELD Excluding International Normalized Ratio Score (MELD-XI) has been associated with poor prognosis in patients with advanced HF [8,9]. MELD-XI can also be used for HF patients with AF receiving anticoagulant therapy which affects INR, and may be applicable even in general HF cases [10,11]. However, data are scarce on the prognostic ability of MELD-XI for CV events, such as cardiac death and hospitalization for worsening HF, as well as in elderly (i.e., ≥65 years old) hospitalized patients with HF. In this context, we have hypothesized that not only history of cardiac disease, brain natriuretic peptide (BNP), and left ventricular (LV) ejection fraction (LVEF), but also multi-organ dysfunction, may affect prognosis in elderly patients with HF. Therefore, the purpose of this study was to examine if MELD-XI could predict adverse CV events in those patients.

## Methods

### Study design

This study was a retrospective analysis from the CURE-HF registry database, which was conducted as a prospective observational investigation of heart failure. A multicenter cohort study was conducted between July 2014 and July 2018 that included 851 hospitalized patients with decompensated HF who were treated at Shinshu University Hospital or its affiliated institutions. The exclusion criteria were acute coronary syndrome, hemodialysis, insufficient data at discharge, and age <65 years. Further exclusion criteria relevant to the analyses included liver disease as diagnosed by viral testing or image testing if performed, such as viral hepatitis, hepatic tumors, cirrhosis, and bile duct disease.

Patient baseline clinical characteristics, laboratory data, and echocardiography results were recorded before discharge. The diagnosis of HF was made by attending clinicians based on symptoms, physical examination, chest X-rays, laboratory data, and echocardiography findings. Hypertension was defined as systolic blood pressure (sBP) >140 mmHg and/or diastolic blood pressure >90 mmHg and the use of antihypertensive agents. Diabetes mellitus was diagnosed as hemoglobin (Hb) A1c ≥6.5%, fasting glucose ≥126 mg/dL, random plasma glucose ≥200 mg/dL, or clinical history of oral hypoglycemic agents and/or insulin use. Dyslipidemia was set as serum total cholesterol ≥220 mg/dL, low-density lipoprotein cholesterol ≥140 mg/dL, or taking lipid-lowering agents. Hyperuricemia was defined as serum urate concentration ≥7.0 mg/dL or the need for antihyperuricemic agents. Patients with sustained ventricular tachycardia (VT) underwent implantable cardioverter defibrillator implantation and/or received antiarrhythmic agents. Ischemic heart disease was judged as patients undergoing percutaneous coronary intervention or coronary artery bypass grafting and/or who were indicated as having significant stenosis (>75%) by coronary angiography. Anemia was determined as Hb <13 mg/dL for males and Hb <12 mg/dL for females according to World Health Organization (WHO) anemia criteria. The selection of possible precipitating factors of HF was made by attending clinicians on admission based on patient interviews, electrocardiogram findings, laboratory data, and echocardiography and coronary angiography results. The precipitating factor of arrhythmia was defined as sustained VT, AF, or any other form of tachyarrhythmia and bradyarrhythmia.

The Ethics Committees of Shinshu University Hospital and each participating hospital approved the study protocol (the approval number: 4237). This investigation was performed according to the Declaration of Helsinki. Written informed consent was obtained from each patient. This study was registered with the University Hospital Medical Information Network (UMIN 000024470).

### Echocardiography

Echocardiography was conducted by an experienced operator in compliance with the recommendations of the American Society of Echocardiography [12]. LV volume and LVEF were measured by the biplane modified Simpson’s method. Estimated systolic pulmonary artery pressure was calculated by adding the estimation of right atrial pressure on the basis of inferior vena cava (IVC) diameter and collapse to the transtricuspid pressure gradient [13]. All echocardiography parameters were performed using standardized equipment (Vivid Ep Ultrasound Machine; GE Healthcare, Chicago, IL, USA).

### MELD-XI

MELD-XI was calculated by the following formula: (5.11 × Ln [total bilirubin] + 11.76 × Ln [creatinine]) + 9.44. The minimum respective values for creatinine and total bilirubin were set at 1.0 mg/dL. The maximum value of creatinine was set at 4.0 mg/dL [8]. We divided the cohort into two groups based on median MELD-XI score: low MELD-XI score (<11) and high MELD-XI score (≥11).

### Clinical outcomes

Patients were followed from admission and evaluated for: 1) the composite of major adverse CV events (MACE) including CV death and re-hospitalization for worsening HF, and 2) non-CV death. Non-CV death included death due to causes such as infection, cancer, hemorrhage, or stroke. Clinical data were collected at the time of scheduled follow-up and by telephone call to each patient. Survival status was ascertained by chart review.

### Statistical analysis

All continuous variables are presented as the mean ± standard deviation if normally distributed, or as the median (interquartile range) for non-normal distribution. Normality was assessed using the Shapiro-Wilk W-test. Continuous variables were compared using the Student’s t-test or Mann-Whitney U test. Categorial variables are expressed as numbers and percentages and compared between groups by means of the Chi-squared test. Kaplan-Meier survival plots were calculated from baseline to the occurrence of adverse events and compared using the log-rank test. Cox proportional hazards regression analysis was used to identify MACE predictors based on clinical characteristics and risk factors. Age, male, and all baseline variables associated with MACE in univariate analysis (p<0.10) were entered into a multivariate model; creatinine and total bilirubin used in the calculation of MELD-XI were excluded. Age and male were included in the model based on regression coefficients to adjust for age and gender effects. The area under the receiver operating characteristic curve (AUC) was employed to compare the abilities of total bilirubin, creatinine, and MELD-XI for predicting MACE. A p-value of <0.05 was considered statistically significant. Statistical analyses were performed using IBM SPSS Statistics version 25 software and EZR software (Saitama Medical Center, Jichi Medical University), which is a graphical user interface for R (The R foundation for Statistical Computing, version 3.6.0).

## Results

### Baseline patient characteristics

**Fig 1:**
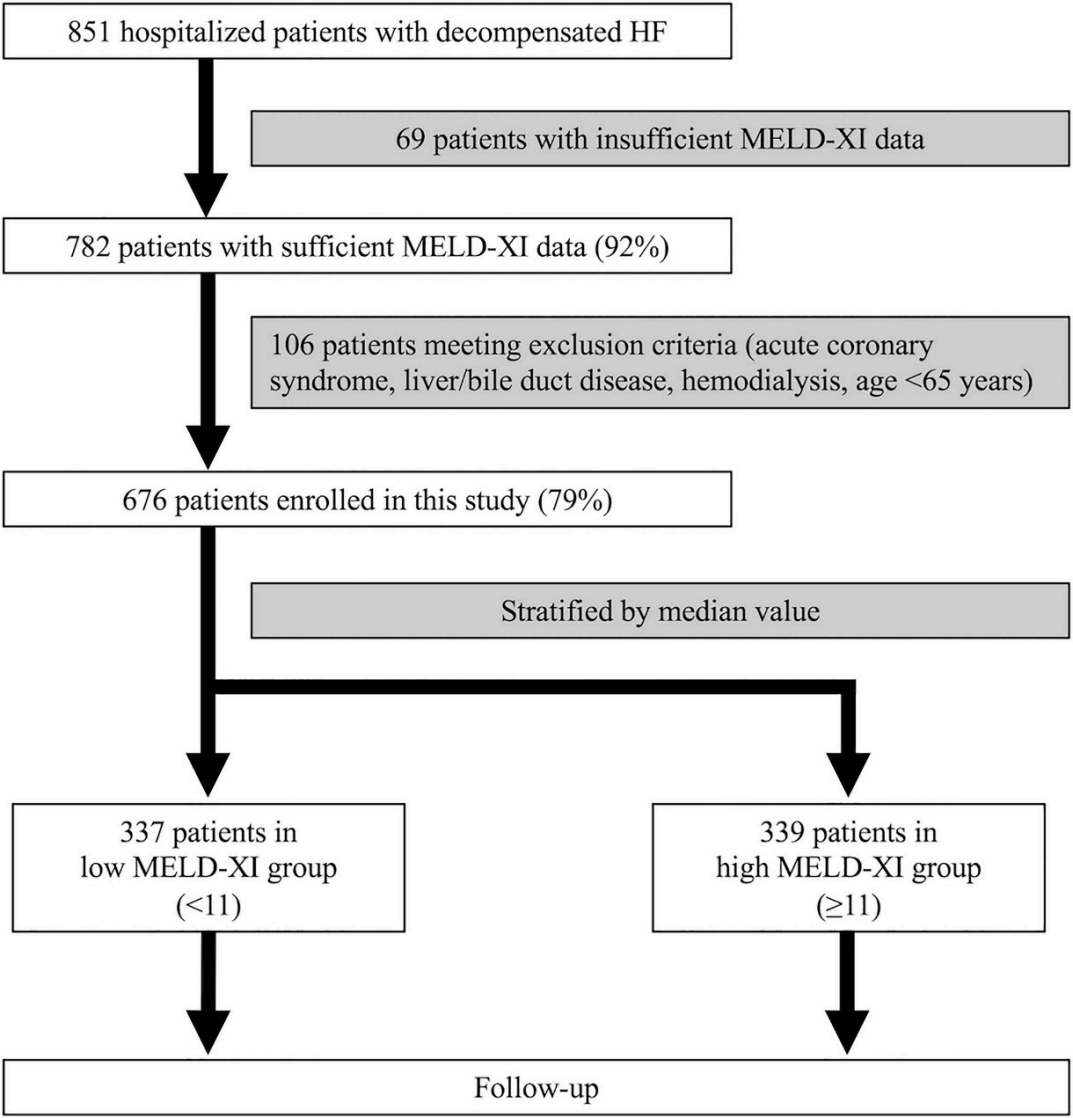
Study design flowchart. HF, heart failure; MELD-XI, model for end-stage liver disease excluding international normalized ratio score.

A total of 676 patients were enrolled in this study (Fig 1). The subjects were divided into the low MELD-XI group (n = 337) and the high MELD-XI group (n = 339) using the median value. The baseline patient characteristics are summarized in Table 1. The median age of the overall cohort was 82 (75, 87) years, and 53.3% were male. The median body mass index was 20.7. Ischemic etiology was prevalent in 27.8% of patients. New York Heart Association (NYHA) function class III or IV was found in 21.2% of patients, and prior HF hospitalization was 35% of patients on hospital discharge. Anemia (64.3%) was the most common comorbidity, followed next by hypertension (63.8%) and AF (54%). Among common precipitating factors of heart failure, arrhythmias were recorded in 30.0% of patients, more than 1 factor was seen in 25.3% of patients, and fatigue from overwork was noted in 22.5% of patients. Angiotensin-converting enzyme inhibitor (ACE-I)/angiotensin receptor blocker (ARB), β-blocker, and mineralocorticoid blocker were prescribed to 69.5%, 69.5%, and 50.6% of patients, respectively. The median BNP was 302 pg/dL and LVEF was 51%.

**Table 1:** Baseline characteristics of study patients according to MELD-XI.

	Overall	Low MELD-XI	High MELD-XI	p-value
		(<11)	(≧11)	
	n = 676	n = 337	n = 339	
**MELD-XI**	11.08 (9.44, 14.13)	9.44 (9.44, 9.44)	14.12 (12.30, 16.86)	-
**Age (yrs)**	82 (75, 87)	82 (75, 87)	82 (76, 87)	0.491
**Male, n (%)**	360 (53.3)	146 (43.3)	214 (63.1)	<0.001
**Body mass index**	20.7 (18.8, 23.4)	20.6 (18.4, 23.3)	21.0 (19.0, 23.6)	0.075
**Systolic blood pressure (mmHg)**	113 (101, 127)	115 (103, 128)	111 (98, 125)	0.006
**Heart rate (bpm)**	69 (60, 80)	70 (61, 80)	69 (59, 79)	0.015
**Ischemic etiology, n (%)**	188 (27.8)	76 (22.6)	112 (33.0)	0.002
**NYHA III/IV, n (%)**	143 (21.2)	55 (16.4)	88 (26.0)	0.002
**Prior HF hospitalization, n (%)**	236 (35.0)	89 (26.4)	147 (43.5)	<0.001
**Comorbidities**				
Hypertension, n (%)	431 (63.8)	216 (64.1)	215 (63.4)	0.856
Diabetes mellitus, n (%)	196 (29.0)	88 (26.1)	108 (31.9)	0.100
Dyslipidemia, n (%)	165 (24.4)	75 (22.3)	90 (26.5)	0.194
Hyperuricemia, n (%)	108 (16.0)	30 (8.9)	78 (23.0)	<0.001
Atrial fibrillation, n (%)	365 (54.0)	175 (51.9)	190 (56.0)	0.283
Ventricular tachycardia, n (%)	38 (5.6)	12 (3.6)	26 (7.7)	0.020
Anemia, n (%)	435 (64.3)	199 (59.1)	236 (69.6)	0.004
COPD, n (%)	39 (5.8)	20 (5.9)	19 (5.6)	0.854
Ischemic heart disease, n (%)	182 (27.0)	76 (22.6)	106 (31.4)	0.010
History of malignant tumor, n (%)	58 (8.6)	35 (10.4)	23 (6.8)	0.095
**Precipitating factors**				
Ischemic events, n (%)	53 (7.8)	27 (8.0)	26 (7.7)	0.604
Arrhythmias, n (%)	203 (30.0)	113 (33.5)	90 (26.5)	0.048
Anemia, n (%)	64 (9.5)	30 (8.9)	34 (10.0)	0.617
Infection, n (%)	97 (14.3)	50 (14.8)	47 (13.9)	0.718
Poor dietary compliance, n (%)	102 (15.1)	43 (12.8)	59 (17.5)	0.089
Poor drug compliance, n (%)	38 (5.6)	17 (5.0)	21 (6.2)	0.516
Fatigue from overwork, n (%)	152 (22.5)	64 (19.0)	88 (26.0)	0.030
Uncontrolled blood pressure, n (%)	79 (11.7)	45 (13.4)	34 (10.0)	0.179
More than 1 factor, n (%)	171 (25.3)	83 (24.6)	88 (26.0)	0.691
**Medications**				
Antiplatelet agents, n (%)	242 (35.8)	106 (31.5)	136 (40.1)	0.017
Anticoagulants, n (%)	391 (57.8)	195 (57.9)	196 (57.8)	0.990
ACE-I/ARB, n (%)	470 (69.5)	229 (68.0)	241 (71.1)	0.375
Beta blockers, n (%)	469 (69.5)	239 (71.1)	230 (67.8)	0.354
Loop diuretics, n (%)	586 (86.7)	286 (84.9)	300 (88.5)	0.165
Tolvaptan, n (%)	143 (21.1)	38 (11.3)	105 (31.0)	<0.001
Mineralocorticoid blockers, n (%)	342 (50.6)	179 (53.1)	163 (48.1)	0.191
Amiodarone, n (%)	46 (6.8)	15 (4.5)	31 (9.1)	0.015
**Laboratory data**				
Hemoglobin (g/dL)	11.6 (10.4, 13.4)	11.2 (10.8, 12.2)	11.4 (9.9, 12.7)	<0.001
Albumin (g/dL)	3.4 (3.1, 3.7)	3.4 (3.1, 3.7)	3.5 (3.1, 3.8)	0.158
Creatinine (mg/dL)	1.10 (0.87, 1.41)	0.90 (0.76, 1.00)	1.41 (1.23, 1.80)	<0.001
Uric acid (mg/dL)	6.9 (5.6, 8.3)	6.4 (5.2, 7.7)	7.3 (6.0, 8.9)	<0.001
Sodium (mmol/L)	139 (137, 141)	140 (138, 141)	139 (137, 141)	0.035
Total bilirubin (mg/dL)	0.64 (0.50, 0.90)	0.64 (0.50, 0.82)	0.63 (0.48, 1.00)	0.150
BNP (pg/dL)	302 (147, 526)	289 (139, 468)	316 (156, 576)	0.160
CRP (mg/dL)	0.23 (0.10, 0. 75)	0.29 (0.10, 0.81)	0.20 (0.10, 0.67)	0.220
Total cholesterol (mg/dL)	159 (134, 182)	162 (142, 186)	151 (129, 178)	<0.001
**Echocardiography**				
Left atrial diameter (cm)	4.5 (4.0, 5.0)	4.4 (3.9, 4.9)	4.6 (4.1, 5.2)	<0.001
LV EDV (mL)	93.0 (63.8, 125.8)	87.4 (58.8, 118.4)	99.9 (70.0, 134.0)	0.001
LV ESV (mL)	42.8 (26.7, 75.3)	39.8 (24.4, 70.8)	47.0 (29.0, 79.6)	0.022
LV EF (%)	51.0 (36.7, 62.9)	51.0 (38.0, 63.1)	51.0 (35.5, 62.0)	0.279
E/e’ (septum)	15.9 (11.8, 20.6)	15.9 (11.6, 21.1)	16.0 (12.1, 20.4)	0.842
E/e’ (lateral)	11.9 (8.5, 16.4)	12.3 (8.6, 16.3)	11.4 (8.5, 16.5)	0.365
IVC diameter (cm)	1.40 (1.16, 1.77)	1.37 (1.16, 1.68)	1.46 (1.16, 1.68)	0.023
TRPG (mmHg)	26.8 (21.6, 34.0)	26.7 (22.0, 34.0)	26.9 (21.0, 34.30)	0.958
SPAP (mmHg)	31.8 (26.2, 39.3)	31.0 (26.2, 38.9)	32.0 (26.3, 40.9)	0.256

Data are presented as the median (interquartile range) or number (%). ACE-I, angiotensin-converting enzyme inhibitor; ARB, angiotensin receptor blocker; BNP, B-type natriuretic peptide; COPD, chronic obstructive pulmonary disease; CRP, C-reactive protein; E, peak early mitral inflow velocity; e’, peak early diastolic velocity at the mitral annulus; IVC, inferior vena cava; LV EDV, left ventricular end-diastolic volume; LV ESV, left ventricular end-systolic volume; LV EF, left ventricular ejection fraction; MELD-XI, model for end-stage liver disease excluding international normalized ratio score; HF, heart failure; NYHA, New York Heart Association; SPAP, systolic pulmonary artery pressure; TRPG, transtricuspid regurgitation pressure gradient.

### Patient characteristics according to MELD-XI

The median MELD-XI was 11.08 (9.44, 14.13). Patients with high MELD-XI were predominantly male. Other variables associated with high MELD-XI included a higher prevalence of NYHA III/IV, history of HF admission, hyperuricemia, VT, anemia, and ischemic heart disease. The values for sBP, heart rate, sodium, and total cholesterol were all significantly lower in patients with high MELD-XI than in those with low MELD-XI, while creatinine levels were higher in patients with high MELD-XI. There were no remarkable differences for age, total bilirubin, or BNP between the groups. The frequencies of precipitating factors in high MELD-XI patients were comparable to those of low MELD-XI patients apart from arrhythmias and fatigue from overwork. The use of an anticoagulant, ACE-I/ARB, β-blocker, or mineralocorticoid blocker was similar between the groups. Regarding echocardiography parameters, higher left atrial diameter, LV volume, and inferior vena cava (IVC) diameter were significantly more frequently observed in patients with high MELD-XI.

During the median follow-up of 1 year (interquartile range: 91–622 days), MACE occurred in 264 patients, which included CV death in 100 patients (HF: 68 patients, sudden death: 10 patients, myocardial infarction: 6 patients, other: 16 patients) and hospitalization for HF in 227 patients. The incidence of CV death and hospitalization for HF was significantly higher in patients with high MELD-XI than in those with low MELD-XI (p = 0.033 and p<0.001, respectively) (Table 2).

**Table 2:** Cumulative incidence of adverse events.

	Overall	Low MELD-XI	High MELD-XI	p-value
	n = 676	(<11)	(≧11)	
		n = 337	n = 339	
**MACE, n (%)**	264 (39.1)	109 (32.3)	155 (45.7)	<0.001
**All-cause death, n (%)**	158 (23.2)	68 (20.2)	90 (26.6)	0.061
**Cardiovascular death, n (%)**	100 (14.8)	40 (11.9)	60 (17.7)	0.033
Heart failure, n (%)	68 (10.1)	25 (7.4)	43 (12.7)	0.039
Sudden death, n (%)	10 (1.5)	5 (1.5)	5 (1.5)	1.000
Myocardial infarction, n (%)	6 (0.9)	4 (1.2)	2 (0.6)	0.688
Other cardiovascular death, n (%)	16 (2.4)	6 (1.8)	10 (2.9)	0.454
**Non-cardiovascular death, n (%)**	58 (8.6)	28 (8.3)	30 (8.9)	0.838
**Hospitalization for HF, n (%)**	227 (33.6)	91 (27.0)	136 (40.1)	<0.001

Cumulative incidence was estimated by the Chi-squared test. HF, heart failure; MACE, major adverse cardiac events (including cardiovascular death and hospitalization for heart failure); MELD-XI, model for end-stage liver disease excluding international normalized ratio score.

### Prediction of MACE

**Fig 2:**
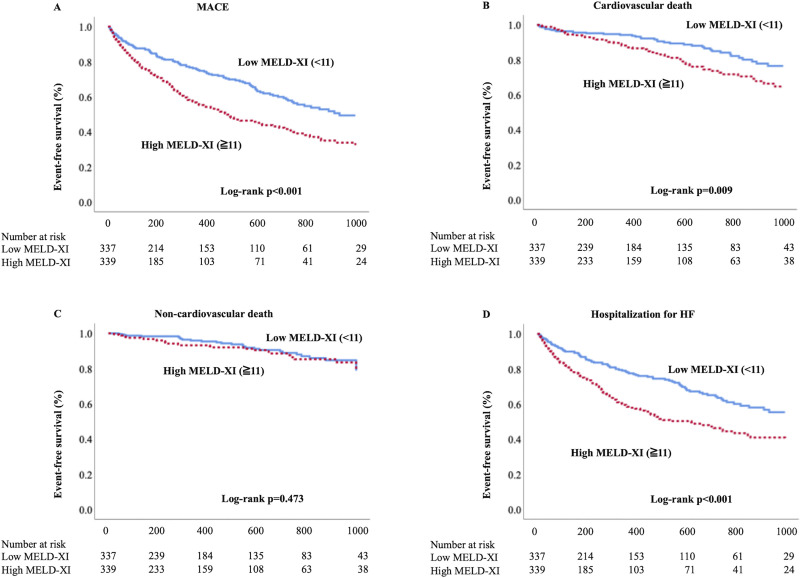
Kaplan-Meier analysis. A) MACE, B) cardiovascular death, C) non-cardiovascular death, and D) hospitalization for heart failure. MACE, major adverse cardiac events (including cardiovascular death and hospitalization for heart failure); MELD-XI, model for end-stage liver disease excluding international normalized ratio score.

Kaplan-Meier survival analysis for events (MACE, CV death, non-CV death, hospitalization for HF) is presented in Fig 2. Patients with high MELD-XI showed a worse prognosis than did those with low MELD-XI (MACE incidence: 45.7% vs. 32.3%, p<0.001). Non-CV death was comparable between the groups.

In the univariate Cox proportional hazard analysis, MELD-XI, age, NYHA III/IV, prior HF hospitalization, sBP, ischemic etiology, VT, anemia, BNP, and LVEF were all associated with higher MACE risk. According to multivariate Cox proportional hazard analysis, MELD-XI predicted poor prognosis, as did age, prior HF hospitalization, VT, and LVEF (Table 3).

**Table 3:** Cox proportional hazards regression analysis of MACE in elderly HF patients.

	Univariate Analysis			Multivariate Analysis		
	HR	95% CI	p-value	HR	95% CI	p-value
**MELD-XI**	1.081	1.050–1.113	<0.001	1.033	1.006–1.061	0.015
**Age**	1.051	1.034–1.068	<0.001	1.060	1.041–1.080	<0.001
**Male**	1.009	0.792–1.287	0.941	1.085	0.813–1.447	0.580
**BMI**	0.985	0.953–1.017	0.356			
**NYHA III/IV**	1.676	1.284–2.189	<0.001	1.114	0.821–1.512	0.487
**Prior HF hospitalization**	2.373	1.862–3.025	<0.001	2.033	1.562–2.645	<0.001
**Systolic blood pressure**	0.990	0.983–0.998	0.015	0.996	0.988–1.003	0.283
**Ischemic etiology**	1.403	1.084–1.816	0.010	1.290	0.968–1.719	0.082
**Atrial fibrillation**	1.020	0.800–1.300	0.873			
**Ventricular tachycardia**	1.870	1.219–2.869	0.008	1.769	1.094–2.859	0.020
**Anemia**	1.590	1.216–2.084	0.001	1.122	0.834–1.511	0.447
**Uric acid**	1.223	0.901–1.661	0.197			
**Sodium**	0.998	0.986–1.010	0.686			
**BNP**	1.000	1.000–1.000	0.036	1.000	1.000–1.000	0.863
**LV EF**	0.992	0.984–1.000	0.051	0.990	0.980–0.999	0.042

BMI, body mass index; CI, confidence interval; HR, hazard ratio. Other abbreviations are as in Table 1.

### Predictive value of MELD-XI

**Fig 3:**
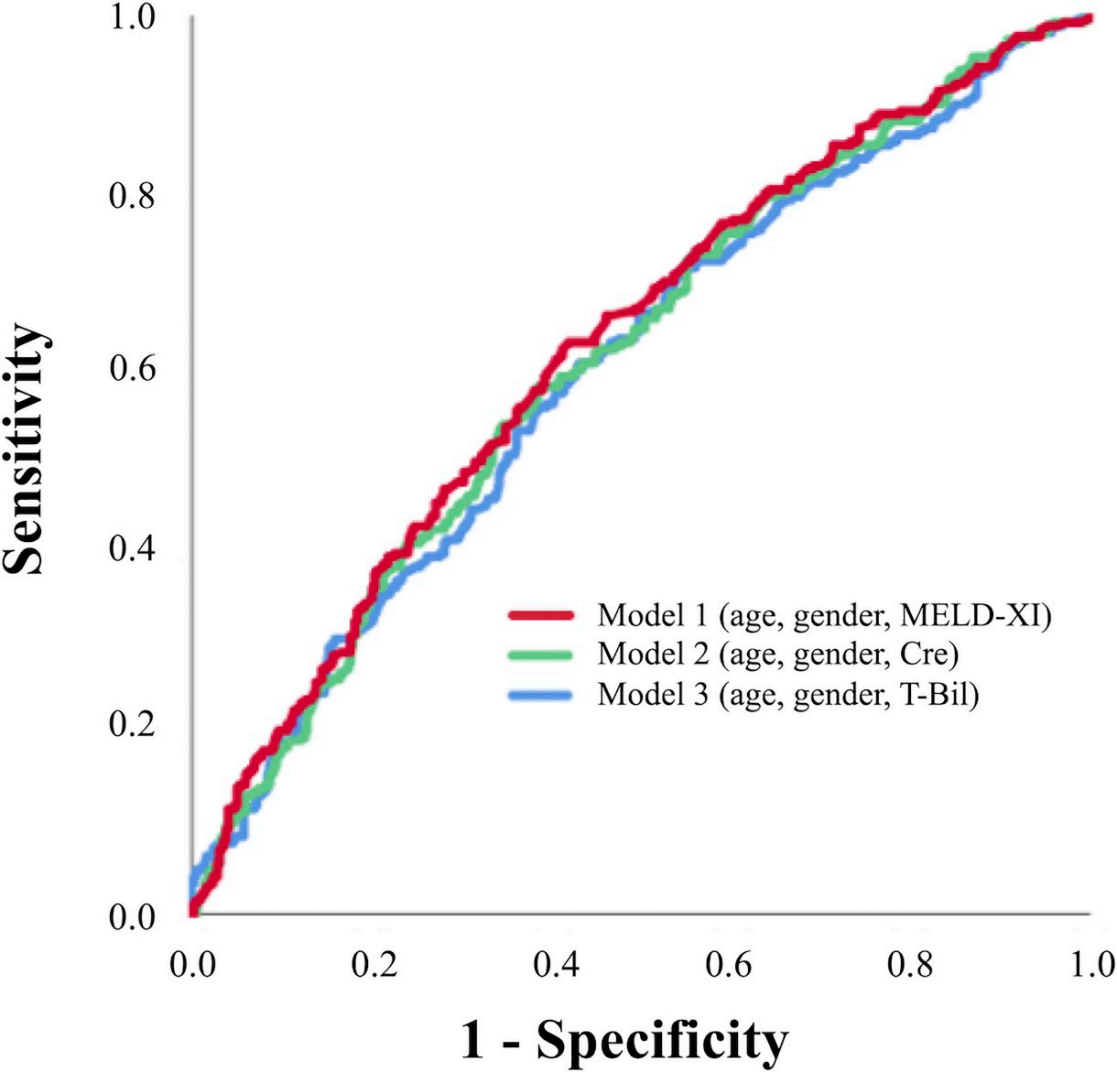
Area under the receiver operating characteristic curve of risk score models for predicting MACE. Cre, creatinine; MACE, major adverse cardiac events (including cardiovascular death and hospitalization for heart failure); MELD-XI, model for end-stage liver disease excluding international normalized ratio score; T-Bil, total bilirubin.

Fig 3 shows the AUCs of 3 risk score models based on creatinine, total bilirubin, and MELD-XI for predicting MACE. The comparison of ROC curves among them to evaluate the discriminatory capacity of each parameter demonstrated that the AUC of MELD-XI was significantly higher than that of either creatinine or total bilirubin (Table 4).

**Table 4:** AUC of risk score model for predicting MACE.

	AUC (95% CI)	p-value
Model 1 (age, gender, MELD-XI)	0.631 (0.588–0.673)	(Reference)
Model 2 (age, gender, creatinine)	0.618 (0.575–0.661)	0.025
Model 3 (age, gender, total bilirubin)	0.608 (0.565–0.651)	0.045

Model 1: -5.271 + 0.049 × age + 0.124 × male (1)/female (0) + 0.064 × MELD-XI. Model 2: -4.766 + 0.049 × age + 0.159 × male (1)/female (0) + 0.21 × creatinine. Model 3: -4.815 + 0.051 × age + 0.194 × male (1)/female (0) + 0.121 × total bilirubin. AUC, area under the receiver operating characteristic curve. Other abbreviations are as in Tables 1 and 3.

## Discussion

This investigation revealed the clinical impact of multi-organ dysfunction using MELD-XI in elderly admitted HF patients. The main findings can be summarized as follows. First, patients with high MELD-XI had significantly more CV events and worse prognosis than did those with low MELD-XI. Second, high MELD-XI was significantly associated with increased CV events independently of age, sex, and widely known predictive factors (NYHA III/IV, previous history of HF hospitalization, BNP, LVEF). These findings indicate that MELD-XI may be a suitable index for stratifying the risk of CV events, and that multi-organ dysfunction diminishes prognosis in elderly admitted HF patients. To our knowledge, this study is the first to report the precise utility of MELD-XI for predicting adverse CV events in aged HF patients.

### Usefulness of MELD-XI

The MELD score has already been validated and is widely used in the risk assessment of patients with advanced liver disease [14–16]. Recently, it has also been implemented in populations with heart disease, including acute HF [17–19]. Kim et al. assessed MELD, MELD-Na, and MELD-XI scores for predicting survival in end-stage HF patients evaluated for heart transplantation [17]. MELD was a good predictor of a diminished outcome, and the presence of serum sodium in the MELD scale improved prognostic strength. However, only MELD-XI could estimate prognosis in patients treated with anticoagulation agents. Those patients receiving anticoagulants tend to have bleeding events and multiple comorbidities, which are risk factors for a poorer outcome. In this study, 57.8% patients received anticoagulation drugs, and 54% exhibited AF. As many elderly HF patients have accompanying AF and are undertaking anticoagulation therapy, MELD-XI may be superior to MELD and MELD-Na in predicting HF prognosis.

Few studies to date have addressed MELD-XI in general HF settings. Abe et al. showed the utility of MELD-XI for predicting all-cause death and cardiac death [10]. Inohara et al. described the usefulness of MELD-XI to stratify the risk of HF re-hospitalization in addition to all-cause death [11]. Similarly in this study, HF patients with high MELD-XI had significantly more frequent cardiac death and HF re-hospitalization. Unlike previous studies, we considered a detailed composite of MACE, which included CV death and re-hospitalization for worsening HF in general HF patients. AUC analysis showed the predictive value of MELD-XI for MACE to be significantly higher than those of creatinine or total bilirubin alone. However, its predictive strength was modest at approximately 0.6. This could have been due to the heterogeneous cohort background containing a variety of conditions, including ischemic heart disease, several types of cardiomyopathies, and AF. If the cohort in this study had been more homogeneous, a higher AUC could have been expected. To the best of our knowledge, the present investigation is the first to show the association of high MELD-XI with increased CV events and poor prognosis in elderly admitted HF patients.

### Heart failure and multi-organ dysfunction

Multi-organ dysfunction in HF is considered a result of simultaneously occurring mechanisms, including low cardiac output, low organ perfusion, sympathetic activation, tricuspid valve insufficiency, right ventricle failure, and increased central venous and intraperitoneal pressure [20–25]. Although this study examined an elderly HF cohort, the median MELD-XI was approximately 11 points, which was similar to previous reports showing MELD-XI scores of 10 to 15 points [10,11,19,26]. Other authors have characterized high MELD-XI as male, previous HF admission, lower systolic blood pressure, and hyponatremia [10,26]. Those factors were in agreement with our results. Right ventricular systolic dysfunction as well as elevated central venous and pulmonary artery pressure have been associated with adverse prognosis in HF [27–30]. In this study, echocardiography showed dilatated IVC diameter and left atrial and LV volume expansion for high MELD-XI, which suggested elevated central venous pressure and cardiac overload, although systolic pulmonary artery pressure did not differ between the groups. Biegus et al. reported that the coexistence of hepatorenal disorder was common (18.5%) in patients with HF in the RELAX-AHF trial [26]. Furthermore, elevated bilirubin and MELD-XI on admission adversely affected prognosis. In the present study, the high MELD-XI group contained higher creatinine, whereas bilirubin was comparable between the groups. This discrepancy may have been caused by the influence of diuretic medication or other treatments at MELD-XI measurement just prior to discharge.

### Precipitating factors of heart failure

In this study, prior HF hospitalization was a potent prognostic factor along with age and MELD-XI. Testa et al. showed that the precipitants of decompensated HF differed between younger and older adults [31]. The elderly subjects of Testa’s cohort and our own frequently had hypertension and AF, used anticoagulants and diuretics, and had preserved LVEF. Arrhythmias and multiple factors were also primary precipitating factors of HF in both studies. Thus, careful management of precipitating factors may help improve outcomes by preventing re-hospitalization for HF.

### Clinical implications

MELD-XI could estimate the prognosis of elderly patients with HF in this study. MELD-XI calculation for predicting CV events is simple and uses routinely measured objective parameters, such as creatinine and bilirubin. The index is also applicable for patients undergoing anticoagulation treatment. Moreover, MELD-XI can be easily acquired on admission and at discharge from admitted patients as well as from outpatients.

In aged patients with HF, the rates of cardiac death and HF re-hospitalization remain high. Although there are presently no established medications or non-pharmacological approaches for such cases, additional management strategies with appropriate guidance and dosage adjustment after discharge may prevent adverse events. Moreover, broader interventions beyond HF management are necessary for those patients, including the treatment of concurrent decompensated chronic conditions, reduction of polypharmacy, monitoring of patient capacity during and after hospitalization to minimize disability, and prescription of physical exercise and nutritional supplementation. An accurate predictive model for CV events will enable tailored medical care according to the patient’s clinical risk factors. As the number of elderly patients with HF is expected to increase, MELD-XI-based risk stratification in addition to ongoing care will play important role in the management of those patients. Clinicians should bear in mind that even if hepatic impairment and pulmonary hypertension are improved at discharge, prognosis may still be poor in patients with prolonged high MELD-XI due to renal function deterioration by diuretics or other medications. Such cases should be followed closely.

### Study limitations

There were several limitations to this study. First, it evaluated MELD-XI at discharge only, and not at admission or hospitalization. Second, RV function was not assessed; however, this test is not routinely performed in general HF patients. Third, since right heart catheterization was performed at varying times in the cohort, right heart volume overload and pulmonary hypertension were not examined at discharge. Fourth, although the usefulness of cardiac metalodobenxylguanidine imaging to improve the prognostic power of the MELD scoring system in patients with HF was reported [32], insufficient scintigraphy results were available in our study. Lastly, as the cohort size was limited, we could not completely eliminate the possibility of selection bias. Nonetheless, MELD-XI score appeared to be a promising and straightforward approach for the risk stratification of CV events in aged patients with HF.

## Conclusions

Cardio-renal and cardio-hepatic interactions estimated by MELD-XI scores may predict future CV events in elderly HF patients.

## Supporting information

S1 Data Original data. (XLSX)
